# 

**Published:** 1853-05

**Authors:** 


*Vicarious examination for a Medical Degree.*— A correspondent of the Western Lancet, writing from Philadelphia, mentions a method of securing a medical degree as singular as it is novel. He says: "A pretty good joke occurred the other day in the faculty of one of our most crowded schools. One of her weaker candidates obtained a student of a neighboring college to *stand the examination* for him, who, it is said, actually run through the duty without detection, until another boy told on him. I have heard of the facility of getting through *in* a crowd; but this is the last instance. No such game, however, need be played here, for *all* may be satisfied with very little trouble."

---

*New York University.*— Dr. Meredith Clymer has resigned the chair of the Theory and Practice of Medicine in the University of New York, and Dr. John A. Swett has been elected his successor. The election of Dr. Swett to this chair will be highly acceptable to the profession. His written productions, and clinical lectures at the New York Hospital, are an earnest of his success in the discharge of the duties of his present responsible position.

---

*Appointment.*— Dr. S. B. Hunt, formerly of Mendon, Monroe Co., N. Y., has been appointed Demonstrator of Anatomy, in the Medical Department of the University of Buffalo, in place of Dr. Hugh B. Vandeventer, resigned. It is due to Dr. Vandeventer to state that his services have been most acceptable to all parties. He leaves the institution with the view of seeking a residence in a southern climate. The warmest wishes of those with whom he has been associated, will go with him. His successor, Dr. Hunt, is an accomplished medical scholar, and we doubt not will prove a valuable acquisition to the institution.

---

*Of the Use of Chloroform in Midwifery.* By Geo. N. Burwell, M. D., one of the Physicians of the Buffalo Hospital.

The paper on the use of Chloroform in Midwifery, read by Dr. Burwell at the annual meeting of the State Medical Society, has been issued in a pamphlet form, making thirty-eight octavo pages. It gives the results in one hundred and eighty cases, with the practical rules for its administration deduced therefrom, and is a valuable contribution to our knowledge bearing on the vexed question of the propriety of anæsthesia in labor.

---

*Meteorological Phenomenon.*—Many of our readers may have met with newspaper notices of a curious meteorological phenomenon lately observed at Louisville, Ky. The Western Medical and Surgical Journal gives the following account of it:

"During a storm of wind and rain, on the night of the 25th of March, a yellow, impalpable powder fell in this city and neighborhood, in such quantities as in some places to cover the ground. Being light, it floated upon the water, and formed a thick film along the sides of the gutters. The phenomenon gains interest from the circumstance that a similar precipitate occurred in Tennessee ten years ago, (March, 1843,) after a cold, backward spring, much like the present. Then, as recently, it was accompanied by a rain which succeeded a day of high southerly winds. The powder had very much the appearance of *flowers of sulphur*. It was combustible, and burnt with the odor of vegetable matter. The explanation generally given of it is, that it is the pollen of flowers wafted into our region by the winds from the South."

---

*Buffalo Hospital of the Sisters of Charity.*—This hospital has been enlarged by the addition of a wing, which will increase its capacity by nearly one-third. The new wards are now ready for occupancy. Vacant lots in front and rear have been purchased, securing for the institution grounds sufficiently ample for quiet, pure air, and any future additions which may be required. The bounty of the state has enabled the trustees to make these important improvements, placing the institution on a firmer basis, and augmenting its charitable resources.

---

*Close of Volume.*—Our readers will take notice, that with the present No. closes the eighth volume of the Buffalo Medical Journal. The index to the volume accompanies this No.

We reserve an announcement of some new arrangements for the next volume until the issue for June.

We shall commence, in our next No. the publication of a valuable monograph on diaphragmatic hernia, by H. I. Bowditch, M. D., of Boston, Mass.

### REPORT ON CHRONIC PLEURISY, BY AUSTIN FLINT, M. D.

THIS Report has been neatly published in a separate form, and may be ordered from the house of DERBY, ORTON & MULLIGAN, Booksellers, Buffalo, N. Y. The price is 50 cents per copy. It can readily be sent by mail.

### NOTICE.

A PRACTITIONER in one of the most eligible country situations in Western New York, contemplating a change of residence to the city, will dispose of house and lot, horse, etc., on favorable terms, to a well educated physician desirous of succeeding him in practice.

Communications may be made through the Editor of the Buffalo Medical Journal.

